# Salivary Metabolomics: From Diagnostic Biomarker Discovery to Investigating Biological Function

**DOI:** 10.3390/metabo10020047

**Published:** 2020-01-26

**Authors:** Alexander Gardner, Guy Carpenter, Po-Wah So

**Affiliations:** 1 Salivary Research, Centre for Host–Microbiome Interactions, Faculty of Dental, Oral & Craniofacial Sciences, King’s College London, London SE1 9RT, UK; alexander.gardner@kcl.ac.uk (A.G.); guy.carpenter@kcl.ac.uk (G.C.); 2 Department of Restorative Dentistry, Dental Hospital and School, University of Dundee, Dundee DD1 4HR, UK; 3 Department of Neuroimaging, Institute of Psychiatry, Psychology and Neuroscience, King’s College London, Maurice Wohl Clinical Neuroscience Institute, London SE5 9RT, UK

**Keywords:** whole-mouth saliva, parotid saliva, gingival–crevicular fluid, submandibular/ sublingual fluid, oral microbiome, metabolic profiling, NMR, MS

## Abstract

Metabolomic profiling of biofluids, e.g., urine, plasma, has generated vast and ever-increasing amounts of knowledge over the last few decades. Paradoxically, metabolomic analysis of saliva, the most readily-available human biofluid, has lagged. This review explores the history of saliva-based metabolomics and summarizes current knowledge of salivary metabolomics. Current applications of salivary metabolomics have largely focused on diagnostic biomarker discovery and the diagnostic value of the current literature base is explored. There is also a small, albeit promising, literature base concerning the use of salivary metabolomics in monitoring athletic performance. Functional roles of salivary metabolites remain largely unexplored. Areas of emerging knowledge include the role of oral host–microbiome interactions in shaping the salivary metabolite profile and the potential roles of salivary metabolites in oral physiology, e.g., in taste perception. Discussion of future research directions describes the need to begin acquiring a greater knowledge of the function of salivary metabolites, a current research direction in the field of the gut metabolome. The role of saliva as an easily obtainable, information-rich fluid that could complement other gastrointestinal fluids in the exploration of the gut metabolome is emphasized.

## 1. Introduction

**Figure 1:**
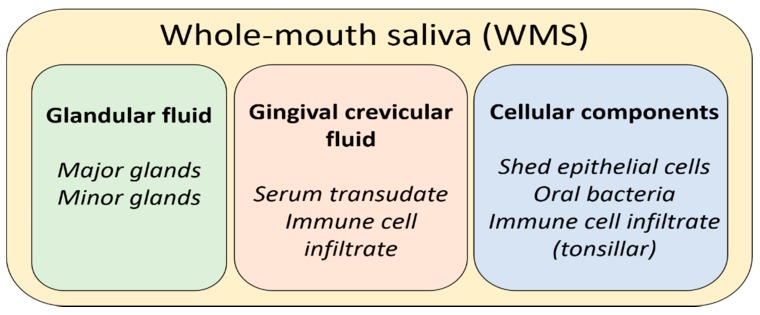
A summary of the net contributions to whole-mouth saliva.

Saliva is a biological fluid produced in the oral cavity by three pairs of major glands and up to one thousand minor glands. It is important to make the distinction between the different fluids that may be described under the umbrella term “saliva”. When referring to the fluid produced upon spitting or drooling, whole-mouth saliva (WMS) would be a more apt descriptor. This reflects the fact that WMS is essentially the net product of major and minor glands, eukaryotic cells (epithelial and leukocytic), bacteria, and gingival–crevicular fluid (GCF) [1]. GCF is a serum-derived filtrate that enters the mouth at the gingival margins of the teeth. While the non-glandular contributions to WMS compose only a fraction of the net fluid volume, their contributions to the net composition are significant. When referring to saliva produced by specific glands, descriptors are used to denote the gland of origin. Fluid produced by the parotid glands is termed parotid saliva (PS), and fluid produced by the submandibular gland and sublingual gland is typically described together as submandibular/sublingual (SM/SL) saliva. This is due to the fact that the submandibular and sublingual ducts share an opening to the oral cavity, making collection of either fluid in isolation extremely impractical, if not impossible. Secretions from minor glands are typically referred to as minor gland saliva, although anatomical descriptors are sometimes applied to differentiate the site (e.g., labial or palatal) [2]. Contributions to WMS from these various sources are depicted in Figure 1.

**Figure 2:**
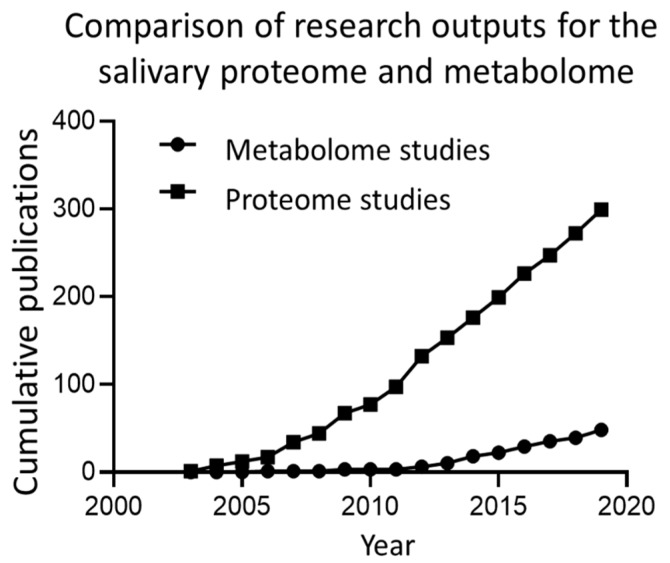
A comparison of research outputs for salivary proteomic and metabolomic studies. Data was gathered by searching Scopus for the terms “saliva” OR “salivary” AND “proteome” OR “proteomics” compared to “saliva” OR “salivary” AND “metabolome” OR “metabolomics” in article title fields.

Salivary composition is complex and variable, both between individuals and temporally within the same individual [3,4]. Salivary protein composition, both of major salivary proteins and low abundance proteins detectable by mass-spectroscopy (MS) proteomics has been comprehensively described elsewhere [5,6,7]. The focus of this review is the metabolite composition of saliva. While some studies have included ions under the umbrella of metabolites [8], this review does not make such an inclusion and focuses on biologically-derived molecules. Until relatively recently, the metabolite composition of saliva has been largely overlooked, or treated as a side-note with respect to protein composition. This discrepancy is illustrated in Figure 2, which shows the research outputs for salivary proteomics and salivary metabolomics, with the current number of metabolomic studies approximating that of proteomic studies one decade ago. Despite this, recent years show a relatively high growth of salivary metabolomic research.

Historically, the study of salivary metabolites has been confined to a select few analytes, often considered in isolation. While this has revealed important knowledge about these metabolites, such as the role of urea in maintenance of salivary pH or lactate in the aetiology of dental caries, systematic metabolite profiling of saliva is a relatively recent avenue of research. Saliva offers many advantages compared to other biofluids. It can be collected safely and non-invasively with minimal training, it is typically produced continually on demand, and it is rich in biological information [9,10]. There are several approaches to obtaining saliva, including the use of proprietary products, e.g., Salivette kits, which are chewable cotton swabs which absorb saliva. Chewing and taste stimulation are both common approaches to increase salivary flow rates, although in practice many studies elect to collect saliva passively, by spitting or drooling into a container as the fluid is produced in the mouth. This may be referred to in literature as unstimulated whole-mouth saliva (UWMS) [2]. The collection of saliva from specific glands can be more complicated. Parotid gland saliva is most commonly collected with the use of a Lashley cup, while submandibular and sublingual gland saliva are typically collected together by pipetting methods and minor gland saliva can be collected using filter papers or capillary tubes [11]. Despite advantages such as ease of collection and preparation, saliva is considerably less studied by metabolic profiling compared to fluids such as urine and plasma, as evidenced by the number of research outputs on the respective fluids [12]. It is unclear why this is the case; however, it could be that a perceived lack of an established literature base would deter researchers unfamiliar with salivary collection. Metabolite profiling of fluids such as urine and plasma were reported by gas chromatography-MS in the mid 1970s [13], and by proton nuclear magnetic resonance spectroscopy (^1^H-NMR) early 1980s [14,15]. Preliminary metabolic study of saliva by ^1^H-NMR was not published until the late 1980s/early 1990’s by which point, study of plasma and urines were established in pathophysiological investigations [16], e.g., of hepatotoxins [17]. The early ^1^H-NMR studies of salivary metabolite composition were performed at low magnetic field strengths by modern standards, leading to relatively low resolution. Additionally, minimal assignments were made to the spectral peaks, partly due to overlapped resonances from macromolecules and insufficient attenuation of the relatively large water resonance [18,19,20]. The earliest ^1^H-NMR study of saliva with comprehensive assignments was that of Silwood et al., 2003 [21]. Subsequent studies, although growing, remain low in number relative to those of other biofluids, and merit discussion in the following section of this review. With respect to MS-based studies, while there is a history of applying the technology to saliva for the targeted detection of analytes such as exogenous drugs and their metabolites, comprehensive profiling of saliva by MS emerged in the early 2010s [22,23]. 

## 2. Approaches to the Study of Salivary Metabolites

Terminology relating to the study of metabolites can be misused, and there are important distinctions between “metabolomics” and “metabonomics”. This review uses the definitions of Nicholson (2006) [24]. Metabolomics is thus defined as “the comprehensive quantitative analysis of all the metabolites of an organism or specified biological sample”, whereas metabonomics is defined as “the quantitative measurement of the multiparametric time-related metabolic responses of a complex (multicellular) system to a pathophysiological intervention or genetic modification”. The key distinction is that the dynamic nature of biological systems in response to stimuli must be considered under metabonomic study. Much of the literature pertaining to saliva would therefore be classified as metabolomics, although a number of studies have begun to address salivary metabolic changes following stimuli such as taste and mastication. The term “metabolic profiling” is also used as a general descriptor for the simultaneous measurement of multiple metabolites in a biological sample. 

There are currently two main technologies used in metabolic profiling—NMR and MS. Both platforms can be further classified. Protons (^1^H) are the most common NMR-active nuclei studied in salivary metabolomics NMR spectroscopy, although other nuclei such as ^13^C are also analyzed in heteronuclear two-dimensional studies. Such an approach is particularly useful in confirming or aiding the assignment of spectral peaks not yet confirmed. As saliva is a liquid, solution state NMR is routinely used, however, solid state NMR has successfully been used to characterize metabolites in salivary calculi [25]. NMR spectroscopy of WMS can be applied with minimal or no sample pre-treatment, although there are advantages to simple steps such as centrifugation to remove cellular constituents. MS analysis of saliva typically involves a metabolite extraction process and a separation step. With respect to saliva, the commonest approach is liquid chromatography MS (LC-MS), although capillary electrophoresis (CE-MS), and gas chromatography-MS (GC-MS) are also used. CE-MS studies are growing in metabolomics research, and this is true for the study of saliva, with the advantages of enhancing the range of polar metabolites detected [26]. MS techniques that do not require a separation step e.g. MALDI (matrix-assisted laser desorption ionization) MS are widely used in salivary proteomics and while such approaches offer promise in metabolomics of other substances, this is not yet common in saliva [27]. Several reviews have been written about the relative advantages and limitations of different MS and NMR platforms in metabolomics [28,29,30,31,32,33]. The combination of platforms to maximize data from metabolomic study has been recognized [32]. This remains rare in salivary metabolomics to date, although a few studies have taken such an approach [8,34]. 

To facilitate metabolomic research of biofluids, protocol papers have been published with the aim of ensuring validity and allowing comparisons between different studies, addressing issues of sample collection, storage, preparation, and analysis. For NMR-based metabolomics, protocols for the analysis of urine and plasma are well established and widely used [35,36]. Consequently, results from subsequent studies conforming to these standardized protocols can be more readily compared. Similar published protocols exist for MS-based metabolomics of these fluids [37,38]. Recommendations for saliva preparation for metabolomic analysis by LC-MS, and more recently, by ^1^H-NMR have been published [12,22]. A lack of protocol homogeneity is a major complication in the meta-analyses of research on salivary metabolomics. 

## 3. Current Knowledge of the Salivary Metabolome

**Figure 3:**
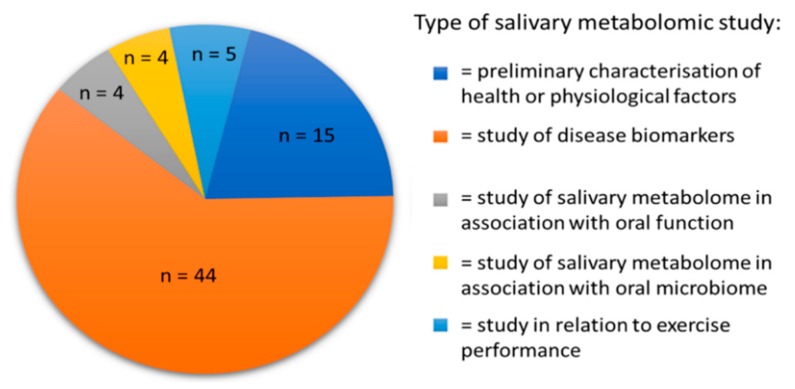
An illustration of the classification of existing original research studies of salivary metabolomics.

There are currently approximately 72 original research articles applying metabolomics to human saliva. These can be broadly classified into different types of study as described in Figure 3. Based on the definitions in the previous section, preliminary studies into salivary metabolites would largely be classified as metabolomic, in that the emphasis was on characterizing and identifying the metabolites that can be measured. Nevertheless, studies looking at the dynamic nature of salivary metabolites have been published and these are increasing in number. This is particularly true as the baseline metabolite composition of saliva in healthy individuals is now fairly well characterized, with the majority of NMR spectral assignments “solved” [8], although there remains debate about certain peak assignments. As seen in Figure 3, most studies of salivary metabolic composition have focused on salivary metabolite profiling as a means of biomarker discovery. Of these studies, eleven have looked at oral cancer, eight periodontal disease and six Alzheimer’s disease. The remainder of conditions studied are limited to one or two studies at present. Oral cancer has the most extensive history of study by salivary metabolomics, as novel diagnostic approaches are sought due to the clinical benefit of early detection [39]. It could therefore be argued that these studies are metabonomic as they introduce the element of analysis in pathological conditions. A list of studies using salivary metabolomics as a source of biomarkers for different oral and systemic diseases is presented in Table 1. 

As indicated in Table 1, the potential for salivary metabolites as diagnostic biomarkers is promising. Many of the studies are self-described pilot or proof of concept studies, featuring relatively low numbers of participants, although participant number does vary with the highest number of cases being 660 Alzheimer’s disease patients and a comparable number of controls. However, implementation of such technologies into clinical diagnostics undoubtedly faces challenges at present. For example, the specificity of metabolites such as valine, alanine, lactate, propionate, and butyrate as disease indicators is questionable. Metabolites such as valine have been found to be upregulated in very different conditions including periodontal disease [40,41] and oral cancer [23,42]. Indeed, Sugimoto et al., (2010) [23] found salivary valine was also increased in breast and prostate cancer, not just oral cancer. Similarly, propionate has been found to be upregulated in a number of diverse pathologies including Alzheimer’s disease [43], dementia [44], periodontal disease [40], and dental caries [45]. Therefore, such molecules may be representative of a non-specific pathological shift in the salivary metabolome. Furthermore, some studies have reported conflicting results. For example, butyrate has been found to be increase or decrease in concentration in association with the presence of dental caries [45,46]. It is likely that single metabolites in isolation may not yield sufficient diagnostic specificity for most disease, however multivariate analyses may offer improved accuracy. 

Further challenges that arise from the current literature base on salivary metabolomics are due to heterogeneity between studies. While the studies listed in Table 1 follow a broadly similar approach in that they attempt to find differences between diseased individuals and healthy controls, there is considerable variation in the, sample collection and preparation methods, analytical platforms used, and statistical methods employed. This has been identified in a recent review of salivary metabolomics for the diagnosis of oral cancer and periodontal disease, where the degree of heterogeneity between studies meant comparison of results from different groups studying the same disease was not possible [47]. For biomarkers to become clinically accepted as diagnostically useful, systemic reviews of large multi-centre trials must be performed. There is therefore a need for both a consensus on sample handling as well as cross-validation of analytical equipment between different centres working on such projects. This is typified by the studies of Wang et al., (2014) published contemporaneously using the same subject groups and analytical techniques, yet recommending different panels of metabolites to discriminate oral cancer from controls [48,49]. 

The studies listed in Table 1, particularly those looking at oral malignancy typically look at discriminating disease from healthy controls. A major challenge in the diagnosis of oral cancer is in determining malignancy from pre-malignant or non-malignant pathology that can have very similar clinical presentations to cancer. Metabonomic studies comparing such conditions remain scarce, although multivariate analysis of data from HPLC/MS analysis of saliva has been shown to discriminate oral squamous cell carcinoma from oral lichen planus and oral leukoplakia, although peak identification was not confirmed [50]. A more recent study also found salivary metabolomics could separate lichen planus from oral cancer, finding discriminatory metabolites upregulated in oral cancer included indoleacetate, putrescine and phosphoethanolamine [51]. Oral cancer and precancerous dysplasia have also been separated from other clinically similar lesions including keratosis and inflammation [52]. 

Further study of similar oral diseases by metabolomics may aid in clarifying pathogenesis and disease classification. For example, recently there have been international changes in the classification of periodontal disease, with aggressive and chronic periodontitis no longer considered separate diseases [53]. Metabolomic evidence supports such a decision from a functional perspective as it was found that there was no metabolomic difference between individuals with aggressive and chronic periodontitis [41]. Salivary metabolomics has also been applied to the study of prognostic biomarkers in periodontal disease following surgical therapy. A shift in the metabolomic profiles of saliva was observed following treatment, indicative of a return from the disease state to a healthy state [54]. Since treatment outcomes from periodontal disease can be difficult to predict the enhanced understanding of the underlying pathology offered by metabolomics is invaluable. 

Ultimately, more work is needed for the translation of salivary metabolomics into useful diagnostic aids. This will be facilitated by the adoption of standardized analytical protocols. The technical aspects of sample preparation and analysis are only one aspect of such protocols. Wider aspects of salivary physiology that might impact on metabolite profile, including timing of collection, choice of stimulus, and participant behaviors must also be considered and steps taken by researchers to standardize these considerations. Prior to characterizing changes in salivary metabolome brought about by disease, it is important to thoroughly characterize the temporal changes in salivary metabolome that occur in health. Work has been conducted to this end, identifying important considerations. 

An important consideration for salivary composition is the timing of sample collection, as salivary composition including total protein is known to display circadian rhythms [84]. MS metabolic profiling showed the majority (85%) of salivary metabolites do not have an apparent circadian pattern, however the remaining 15% of salivary metabolites exhibit circadian effects [85]. The majority of these circadian-regulated metabolites are amino acids, with tyrosine and arginine, for example, appearing to mirror the circadian pattern of total protein concentration. Circadian effects gave also been demonstrated by NMR metabolic profiling [86]. The NMR salivary metabolite profile has also been shown to vary more within a day than between days, although the flow rate of saliva was demonstrated to be independent from the metabolic variations [87]. Nevertheless, the intra-individual variation of salivary metabolite profile both within a day and between days has been shown to be considerably lower than inter-individual variation [88], indicating salivary metabolites can discriminate between individuals. Importantly, the first saliva collected on waking was found to be significantly richer in many metabolites which may represent changes in saliva production during sleep [88]. Salivary metabolic content has also been found to be independent of diet, as dietary standardization can reduce inter-individual variation in the metabolic composition of urine, but a similar reduction was not observed with saliva [89]. 

The metabolic composition of saliva is also influenced by stimulation and certain participant behaviors. Stimulation using citric acid caused a decrease in the majority of metabolites measured [90,91]. While this decrease is largely attributable to dilution due to increased fluid content of the saliva, metabolites were not diluted proportionally, indicating metabolite specific effects [91]. Stimulation by chewing also appears to cause contrasting changes in metabolite concentration compared to citric acid stimulation. Whereas citric acid stimulation reduced the concentration of metabolites, chewing has been demonstrated in both MS and ^1^H-NMR studies to cause an increase in the concentration of the majority of metabolites. This was attributed to the mechanical action of chewing liberating metabolites from oral biofilms [92,93]. A large MS study of physiological and environmental factors demonstrated salivary metabolite profile is not significantly affected by lifestyle factors such as alcohol consumption, fasting, oral hygiene, medication use or nutritional supplementation. The largest effects were observed for sex, followed by collection method and then smoking. Body mass index (BMI) was shown to have an effect only when comparing underweight individuals to healthy weight, but not when comparing overweight individuals. Menstrual status had no effect when considering female participants [94]. ^1^H-NMR analyses have found salivary metabolite profile is independent of sex and BMI when considering net metabolite profile [86], although sex-based metabolic differences have been found when quantifying individual metabolites [91]. Collection method is another caveat for the metabolomic study of saliva, with the use of collection systems as opposed to passive drooling being identified as a potential source of exogenous analytes in samples [46]. 

## 4. Role of Host–Microbiome Interactions in Modifying Salivary Metabolic Composition

**Figure 4:**
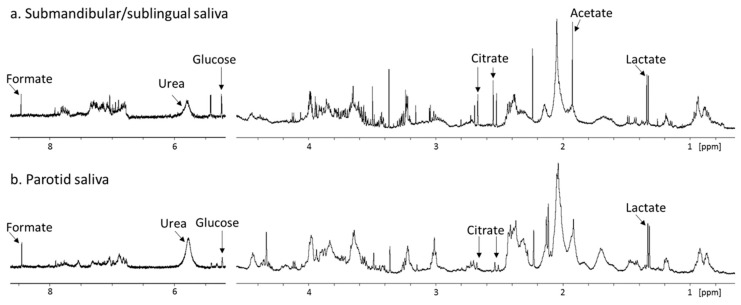
Partial 600 MHz 1D ^1^H-NMR spectra (δ0.7 to 8.5 ppm, excluding δ4.5 to 5.5 ppm), comparing **a**. submandibular/sublingual saliva and **b**. parotid saliva from the same donor. Prominent peaks are labelled. Samples were collected and spectra were acquired on separate occasions as previously described [95]. Spectra are scaled to the lactate peak to assess relative metabolite concentrations. The acetate in a. reflects some cross-contamination with WMS during collection as submandibular/sublingual saliva was pipetted from the floor of the mouth upon secretion, inevitably contacting the oral cavity briefly.

The role of oral microbial communities in shaping the salivary metabolome has been speculated in literature, and the need for further research into this area has been identified. Only recently is the true extent of microbial contributions to WMS metabolite composition becoming appreciated. This was demonstrated by comparison of sterile parotid gland saliva with WMS, which reveals that prior to entering the oral cavity, glandular saliva is devoid of many WMS metabolites such as acetate, butyrate and propionate, collectively known as short-chain fatty acids (SCFAs). Furthermore, the concentration of many WMS metabolites including SCFAs, amines, phenylalanine, glycine, and succinate correlate strongly with the bacterial load in WMS [95]. Such a finding has significant implications for the diagnostic utility of these metabolites, as highlighted in the previous section, as certain metabolites that have been identified as putative biomarkers may be reflective of changes in the oral microbiome. These may not reflect a specific disease process but, for example, a general impairment of oral hygiene for various reasons, i.e., pain or discomfort in oral cancer and periodontal disease or reduced physical and mental ability to care for oneself in cognitive diseases. Host-derived salivary metabolites include lactate, citrate, and urea. Urea has been shown to negatively correlate with salivary bacterial load as well as dental plaque as it is utilized by oral bacteria [60,95]. Previous metabolomic study of PS has been complicated by low spectral resolution or low number of donors [20,96]. ^1^H-NMR spectra of SM/SL saliva have also been published but these suffer from the same low resolution as well as the presence of sialadenitis (gland inflammation) in the donor [20]. Figure 4 shows a comparison between PS and SM/SL saliva collected from the same donor. The spectra are highly similar indicating that there are no major differences in metabolite composition of saliva collected from the different major glands. Another major salivary metabolite of host origin is taurine. Taurine is an abundant metabolite in many animal tissues [97], although its entrance into saliva has remained unclear, particularly as salivary levels exceed circulating plasma levels despite taurine being absent from glandular saliva. Taurine is present at high concentrations in GCF and is likely concentrated via immune cells which have been shown to be capable of concentrating up to 50 mM taurine [98]. 

As well as contributing heavily to the net metabolic composition of WMS at rest, the oral microbiomes will also rapidly alter salivary metabolite composition upon exposure to fermentable carbohydrates. This process has been known for eight decades and was proved by the use of traditional biochemical methods [99]. More recently, metabolomics has been used to visualize this process in dental plaque [100,101]. The changes seen in plaque following exposure to glucose are also mirrored in saliva following exposure to sucrose. Although ^1^H-NMR metabolomics does not have the sensitivity to detect certain glycolysis pathway intermediates, salivary metabolites such as lactate, pyruvate, succinate, acetoin, and alanine have been observed to increase after exposure to sucrose [12]. There is also preliminary evidence for the intra-oral breakdown of other exogenous substances including non-nutritive sweeteners. Following oral exposure to aspartame, salivary aspartate and phenylalanine concentrations (the constituent amino acids of aspartame) increased. Whether this is due to host or microbial peptidases is not clear [90].

A further area lacking in knowledge at present is the substrate for oral microbes. For example, acetate, which is the most concentrated salivary metabolite has been previously speculated as resulting from degradation of either dietary carbohydrates [8] or collagen and amino acid catabolism in the absence of carbohydrate sources [40]. One of the major differences between the gut and oral microbiomes, and their respective metabolomes is the availability of exogenous nutrients. In the oral cavity this is transient in most individuals, and saliva clears carbohydrates from the oral cavity by its physical and enzymatic actions typically within an hour [102,103]. In the colon however, undigested fermentable nutrient sources are a more constant presence. Nevertheless, salivary metabolites are generated in the absence of exogenous carbohydrate as evidence by the fact that salivary metabolites on waking are generally more concentrated than at other times throughout the day [88]. As salivary flow rate was not reported by Wallner-Liebmann et al. (2016) [88], some of this concentration increase may simply be due to a reduced salivary flow on waking compared to other times of the day [84]. The metabolite concentration increases were not uniform, however, suggesting genuine changes in metabolite production. The largest fold changes in metabolite concentration on waking relative to later in the day were 15.7, 6.0, 3.6, 3.5, and 3.1 for valine, trimethylamine, butyrate, methylamine, and choline, respectively. Large increases in free valine are suggestive of proteolysis, and butyrate production may represent the terminal metabolite in a range of amino-acid catabolic pathways, particularly lysine [104,105]. Methylamine and trimethylamine are products of microbial choline metabolism [106], and choline itself may be increased due to reduced salivary clearance of shed buccal epithelial cells during sleep [12]. It is appreciated that salivary proteins are a source of nutrients for oral bacteria although this is also recognized as an area of limited study [107]. Recently it has been shown that oral microbial biofilms will catabolize salivary proteins, generating abundant SCFAs, proline, 5-aminopentanoate, and putrescine. Consumption of succinate and lactate from the baseline sterilized saliva was also observed [108]. 

## 5. Potential Physiological Significance of Salivary Metabolites

To date, the physiological significance of salivary metabolites is largely unknown. Unlike in the colon, where bacterial generated metabolites such as SCFAs, lactate and succinate are known to have important roles in host health, equivalent function for these same metabolites in the oral cavity are unknown. SCFAs are known to have an important role in maintaining epithelial integrity in the gut. This is particularly true for butyrate, which upregulates tight junction expression and regulates proliferation of colonic epithelial cell, via histone deacetylase inhibition, upregulating expression of genes promoting apoptosis [109,110]. Gut SCFAs have also been demonstrated to have immune regulation functions, metabolic functions, and to play a role in regulation of host satiety and appetite [109,111,112]. These functions are typically mediated by the actions of SCFAs on G-protein coupled receptors (GPCRs) [111,113]. Additional microbial-derived metabolites in the gut have been shown to exert physiological functions via their role as signaling molecules [111]. These include succinate and lactate, both of which are present in saliva. Succinate is traditionally considered a citric-acid cycle intermediate although recently evidence is emerging to support its role as regulator of gluconeogenesis as well as having anti-inflammatory effects [114]. Lactate has similarly been long considered a metabolic waste product, although evidence for diverse physiological functions is emerging including immune regulation, anti-inflammatory properties and maintenance of epithelial barriers [115]. Enabling cross-feeding between different microbial species is a further important function of metabolites such as succinate and lactate [116]. 

Given the growing appreciation of the interaction between gut metabolites and host behaviors such as taste and nutrient detection [112], modulation of taste receptors by microbial products has been proposed [117]. Such a hypothesis has not yet been directly investigated in humans, although associations between salivary metabolomics and sensory perception have been investigated. The most convincing evidence to date is concerning the sensory perception of oleic acid. It has been demonstrated that, compared to sensitive oleic acid perceivers, insensitive oleic acid perceivers have higher concentrations of salivary acetate and butyrate and lower concentrations of lysine and fatty acids [118]. Furthermore, differences in salivary metabolite composition following oral exposure to oleic acid were observed between sensitive and insensitive oleic acid perceivers. Sensitive perceivers were characterized by increases in acetate, propionate, formate, lysine, valine and GABA, whereas insensitive perceivers were characterized by increases in galactose, glucose, lactate, threonine, phosphocholine, and ethanolamine [119]. This evidence cannot be used to infer a causal relationship between salivary metabolites and sensory perception. Given the strong evidence linking many of these metabolites to oral microbial activity, however, these studies do present a solid evidence base to support the hypothesis that oral microbial activity is related to host oral nutrient sensing. There is additional evidence of an association between other salivary metabolites and sensory perception. These include an inverse association between salivary glucose and sweet sensitivity [120], although this was only found in female participants, and a similar relationship between salivary urea and bitter taste sensitivity in renal disease participants [121]. While glucose tastes sweet and urea tastes bitter, it is unlikely salivary levels would approach the concentrations necessary to be perceptible and directly mask exogenous tastants [122,123]. Both molecules, however, do represent endogenous salivary nutrients that will be consumed by oral bacteria to different extents, so may serve as useful markers of net oral microbial function. 

Another interesting area of salivary metabolomics that is emerging is in sports and exercise physiology. Saliva represents an ideal fluid in the monitoring of athletic performance, where repeated sampling of blood may be excessively invasive or even detrimental to performance. Traditionally one of the best studied salivary analytes in this regards is lactate, often assayed in isolation. Plasma lactate is widely used in sports medicine as an indicator of performance and training intensity, as it is accumulates during anaerobic respiration when the rate of lactate production exceeds the rate of removal [124]. Salivary lactate levels have been shown to closely correlate with plasma levels following exercise in both short- and long-distance runners [125,126]. Furthermore, exercise-induced increases in salivary lactate are reproducible and independent of post-exercise reduction in salivary flow rate [125]. Recently, salivary metabolomics is identifying new metabolic markers of exercise tolerance and sports performance. Metabolites indicative of skeletal muscle use and muscle catabolism such as 3-methylhistidine and taurine have been putatively identified [127], with the latter being found to correlate with distance travelled by soccer players [128]. Several other salivary metabolites have been found to reflect performance following standardized bouts of exercise. These include metabolites associated with increased energy demands such as creatine, inositol, glucose, citrate and lactate [129]. Other metabolites of less obvious functions in man such as δ-valerolactam (2-piperidone) have also been implicated in changing post-exercise [130]. It was speculated that δ-valerolactam could be a product of cadaverine metabolism, a known lysine degradation product in saliva. Recently recommendations were made to address challenges of using salivary metabolomics in association with exercise. A major challenge is in accounting for coinciding changes in fluid volume brought about by exercise. Approaches to account for this effect included normalizing metabolite concentration to either salivary total protein or osmolality. Both approaches were believed to have merit, with neither being definitively recommended. An important recommendation was regarding timing of sample collection, which should be without delay upon cessation of exercise [131]. 

Little is known about how salivary SCFAs interact with oral epithelial cells. It has been shown that in TR146 cells (a squamous cell carcinoma derived cell line commonly used to model buccal epithelium), acetate and lactate have an inhibitory effect on cell growth. This was only observed at relatively high concentrations, 20–30 mM acetate, and 10–20 mM lactate [132]. These concentrations are an order of magnitude higher than those typically found in saliva. Propionate and butyrate have been studied in association with gingival fibroblasts. Butyrate conferred a dose-dependent inhibitory effect on gingival fibroblasts at concentrations of 2–4 mM. Propionate also inhibited gingival fibroblasts at concentrations of 4–8m mM, however, growth inhibition was not seen for all donor samples, suggesting a less generalized inhibitory effect than that of butyrate [133]. It is possible that in cases of severe periodontal disease, butyrate and propionate concentrations would exceed these minimum in-vitro inhibitory concentrations [134]. Taurine has also been studied in association with periodontal disease. Epithelia from excised gingival tissue have been shown to grow more efficiently on collagen membranes hydrated with 1% taurine than on control collagen membranes [135]. Furthermore, daily oral administration of 500 mg taurine has been shown to improve both circulating and local redox status (within gingival pockets) as well as improve clinical indicators of periodontal disease within fifteen days [136]. 

Recent evidence has implicated salivary citrate in association with the physical properties of saliva [137]. Whether there is a causative role for citrate in contributing to the extensional rheology of saliva is at present unclear, as is any precise mechanism. Other salivary analytes associated with salivary rheology include calcium and bicarbonate [138], and it is known that calcium chelation is an important step for the unpacking of salivary glycoproteins (viscoelastic macromolecules) following secretion [139,140]. It is possible that the interactions between calcium, citrate, bicarbonate, and glycoproteins at the point of salivary secretion ultimately determine the net physical properties of the resulting fluid. Alternatively, salivary citrate may simply be a product of gland stimulation alongside other constituents such as mucin but is not otherwise involved in mediating the physical properties. 

## 6. Future Directions for Salivary Metabolomics

Omics technologies can be thought of as providing stratified degrees of biological information. Genomic analysis summarizes the potential genetic information present in a biological system. Transcriptomics and proteomics can then reveal the translation of this biological information into biomolecules with functional potential. However, only metabolomics will provide information on the net function of transcribed, translated and modified proteins within the biological system being studied [141,142]. Gene sequencing technologies previously revolutionized oral microbiology, greatly enhancing the information gained about the microbial species occupying the oral cavity, up to half of which are unculturable [143]. In a similar fashion, metabolomic analysis may represent the next paradigm shift in oral microbiology, with the functional information gained about these complex microbial communities being identified as a key area of future study [104]. This concept has been demonstrated as valid and useful in the study of how the gut metabolome reflects the gut microbiome [144].

At present, very few studies have studied saliva using both sequencing and metabolomics technologies. The most comprehensive study of this type has made steps in elucidating the relationships between salivary metabolites and associated microbial species [145]. There is a clear need for future work to reconcile the functional information provided by metabolomics with the oral microbial composition and host factors that may modify the latter. Therefore, further studies with a combined analytical approach will be essential in future. Another aspect of salivary metabolomics that will merit further study is concerning the dynamic nature of the metabolic events that occur in the mouth. Currently, the majority of studies analyze a single sample from each participant which essentiality creates a static “snapshot” of the present biochemical activity. While some studies have analyzed multiple samples from participants, usually with the aim of characterizing the temporal stability and reproducibility of salivary metabolite profiles, sampling intervals are typically of the order of hours [87,88]. Preliminary studies have shown that significant metabolic changes occur in the order of seconds when the oral environment is exposed to changes brought about by activities such as nutrient intake or oral hygiene. This has been demonstrated both in-vitro with several single species biofilms [100], and in-vivo [101]. A combination of in-vitro and in-vivo work is a useful approach to breaking down the complexity of the oral metabolome, as controlled environments can be created in-vitro, allowing investigation of events that would otherwise be obscured in-vivo. 

**Figure 5:**
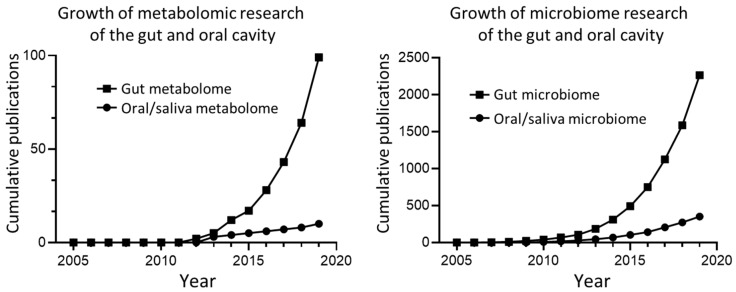
A comparison of the cumulative publications for metabolomics and microbiome studies of the gut and oral cavity. Data was collected from Scopus by searching publications with “gut metabolome”, ”gut microbiome”, ”oral OR saliva metabolome”, and ” oral OR saliva microbiome” in the title field. Note that the total publications for microbiome studies are considerably greater than metabolome studies for both the gut and oral cavity.

A final consideration for future metabolomic studies is the cross-disciplinary study of multiple biofluids. This is particularly prudent in regard to saliva, given one of the main advantages of studying saliva is the ease of collection. Much of the knowledge of the human microbiome, microbial metabolome and resulting consequences for host health stems from study of the gut, with equivalent oral study remaining minimal, (Figure 5). The oral cavity and gut represent portions of the same biological system, and there would almost certainly be great benefit in enhancing how the metabolic activity of these microbial communities relates to each other. For example, if it could be demonstrated that certain disease-related dysbiotic changes in the gut are reflected in oral cavity then this finding would simplify diagnosis in the future. 

## 7. Conclusions

Salivary metabolomics is gaining recognition as a valuable source of biological information. Of particular importance is the fact that much of the metabolite content of saliva is derived from the oral microbiome. Although the majority of metabolites that are detectable in saliva by ^1^H-NMR have been identified, there remains considerable work in interpreting the significance of these different metabolites. It is anticipated that future work concerning the salivary metabolome will focus on determining whether salivary metabolites possess functional roles and deepening the understanding of the relationship between metabolome and microbial function. Researchers working on metabolomics of other biofluids should be encouraged to consider whether the complementary use of saliva could augment their work. This is pertinent as saliva is easy to collect and the collection, handling and preparation of saliva for metabolomic analysis has been fairly well researched and is relatively simple. 

## Figures and Tables

**Table 1:** A summary of studies investigating the potential of salivary metabolomics in biomarker discovery.

Disease (n. Disease vs. n. Control)	Metabolomic Technique	Biomarkers Identified (Raised/Lowered in Disease)	Reference
*Oral Cancer (69 v. 87))*	*Capillary electrophoresis time-of-flight mass spectrometry (CE-TOF-MS)*	Alanine, taurine, leucine/isoleucine, histidine, valine, tryptophan, glutamate, threonine, carnitine, pipecolic acid	[23]
(24 v. 44)	*CE-TOF-MS*	3-phosphoglycerate, carnosine, phosphoenolpyruvate, dihydroxyacetonephosphate Pipecolate, s-adenosylmethionine	[55]
(37 v. 34)	*Ultraperformance liquid chromatography coupled with quadrupole-TOFMS (UPLC-QTOFMS)*	Lactate, n-Eicosanoic acid Valine, γ-aminobutyric acid (GABA), Phenylalanine	[42]
(30 v. 30)	*Reverse phase liquid chromatography and hydrophilic interaction chromatography with TOF-MS*	Propionylcholine, succinic acid, lactic acid Acetylphenylalanine, carnitine, phytosphingosine	[48]
(101 v. 35)	*^1^H-NMR and LC-MS/MS*	Glycine, proline	[34]
(22 v. 18)	*UPLC-QTOFMS*	1-methylhistidine, inositol 1,3,4-triphosphate, dglycerate-2-phosphate, 4-nitroquinoline-1-oxide, 2-oxoarginine, norcocaine nitroxide, sphinganine-1-phosphate, pseudouridine L-homocysteic acid, ubiquinone, neuraminic acid, estradiol valerate	[56]
(22 v. 18)	*CE-MS*	Choline, hydroxyphenylacetic acid, 2-hydroxy-4-methylvaleric acid, valine, 3-phenyllactic acid, leucine, hexanoic acid, octanoic acid, terephthalic acid, γ-butyrobetaine, 3-(4-hydroxyphenyl)propionic acid, isoleucine, tryptophan, 3-phenylpropionic acid, butyric acid, cadaverine, 2-oxoisovaleric acid, trimethyllysine, taurine, glycolic acid, 3-hydroxybutyric acid, heptanoic acid, alanine Urea	[57]
(7 v. 7)	*LC-MS/MS, LC-MS, GC-MS*	Guanosine-3-monophosphate, N-acetylputrescine, 2-Hydroxyglutarate, Adenosine-3-monophosphate, Uracil, Spermidine, 3-Glycosyl tryptopha	[58]
*Oral Leukoplakia* (32 v. 34)	*UPLC-QTOFMS*	Isoleucine, phenylalanine, threonine, homocysteine, n-tetradecanoic acid, 4-methoxyphenylacetic acid	[42]
*Breast Cancer* (30 v. 87)	*CE-TOF-MS*	Alanine, leucine/isoleucine, valine, glutamate Taurine, Threonine	[23]
(124 v. 42)	*CE- and LC-MS*	Polyamines, spermine	[59]
*Prostate Cancer* (18 v. 87)	*CE-TOF-MS*	Alanine, leucine/isoleucine, histidine, valine, tryptophan, glutamic acid, threonine, carnitine Taurine	[23]
*Periodontal Disease* (11 v. 87)	*CE-TOF-MS*	Alanine, leucine/isoleucine, valine, tryptophan, threonine Pipecolic acid, taurine	[23]
(21 v. 22)	*Proton nuclear magnetic resonance spectroscopy (^1^H-NMR)*	Acetate, GABA, propionate, *n*-butyrate, succinate, trimethylamine, valine Pyruvate, *n*-acetyl groups	[40]
(n = 909, significant correlations between [metabolite] and disease severity)	*Ultra-high performance liquid chromatography and tandem MS (UHPLC-MS/MS)*	Phenylpropionate, phenylacetate	[60]
(50 v. 50)	*GC-MS and LC-MS*	Hydroxyeicosatetraenoic acid, prostaglandins, isoprostanes, thromboxane Hydroxyoctadecadienoic acid, prostacylcin	[61]
(62 v. 52)	*^1^H-NMR*	Alanine, valine, leucine, glutamate, succinate, lactate Pyruvate	[62]
(30 v. 15)	*^1^H-NMR*	Methanol, acetone Taurine, lactate	[63]
(26 v. 25)	*^1^H-NMR*	Butyrate GABA, lactate, methanol, threonine	[64]
*Periodontal Disease* *(Aggressive)* (28 v. 39)	*^1^H-NMR*	Isoleucine, proline, valine, tyrosine, phenylalanine Lactate, pyruvate, *n*-acetyl groups	[41]
*Periodontal Disease* *(Chronic)* (33 v. 39)	*^1^H-NMR*	Formate, phenylalanine, tyrosine Sarcosine, Lactate, pyruvate, *n*-acetyl groups	[41]
*Dental Caries* (15 v. 18)	*^1^H-NMR*	Propionate, butyrate Saccharides	[45]
(15 v. 23)	*^1^H-NMR*	Extensive list of amino acids and organic acids Butyrate, acetone	[46]
*Primary Sjögren’s Syndrome* (12 v. 21)	*Gas chromatography* *-MS (GC-MS)*	39 metabolites including organic acids, amino acids and carbohydrates	[65]
(15 v. 15)	*^1^H-NMR*	Alanine, glycine, butyrate, taurine, phenylalanine, choline, tyrosine	[66]
*Dementia* (10 v. 9)	*CE-TOF-MS*	Tyrosine, arginine	[67]
(17 v. 34)	*^1^H-NMR*	Acetate, histamine, propionate Dimethyl sulfone, glycerol, succinate, taurine	[44]
*Alzheimer’s Disease* (256 v. 218)	*Faster ultra-performance liquid chromatography-MS (FUPLC-MS)*	Sphinganine-1-phosphate, ornithine, phenyllactic acid Inosine, 3-dehydrocarnitine, hypoxanthine	[68]
(32 v. 45)	*LC-MS*	Histidinyl-Phenylalanine, methylguanosine, choline-cytidine	[69]
(9 v. 12)	*^1^H-NMR*	Acetone, propionate	[43]
(25 v. 25)	*Flow injection analysis- tandem mass spec. (DFI-MS/MS)*	acyl-alkyl phosphatidylcholines	[70]
*Alzheimer’s Disease (relative to mild cognitive impairment;* (660 v. 586)	*(FUPLC-MS)*	Sphinganine-1-phosphate, cytidine, pyroglutamic acid, L-glutamic acid, L-tryptophan, ornithine, phenyllactic acid 3-dehydrocarnitine, hypoxanthine, Inosine	[71]
*Mild cognitive impairment relative to health* (20 v. 20)	*LC-MS*	Taurine, several putative di- and tripeptides	[72]
*Parkinson’s disease* (76 v. 37)	*^1^H-NMR*	Histidine, tyrosine, phenylalanine, acetate, n-acetyl groups, propionate, Isoleucine, lysine, glycine, valine, GABA, fucose, trimethylamine, trimethylamine-oxide, alanine, acetoin	[73]
*Celiac disease* (13 v. 13)	*GC-MS*	Carbon disulfide, 1-chlorodecane, trichloromethane Alcohols, phenols, ketones	[74]
*Sarcoidosis* (24 v. 45)	*^1^H-NMR*	Lactate, acetate Methanol, n-acetyl groups	[75]
*Recurrent aphthous ulceration* (45v. 49)	*LC-MS/MS*	Tryptophan, 5-methoxytryptamine, 5-methoxytryptophan Estrone sulfate, 17β-Estradiol 3-sulfate, Dehydroepiandrosterone sulfate	[76]
*Untreated HIV* (21 v. 8)	*^1^H-NMR*	Butyrate, Lactate	[77]
*Treated HIV* *(Antiretroviral therapy)* (12 v. 8)	*^1^H-NMR*	Butyrate, Lactate, propionate	[77]
*Hepatitis B* (20 v. 16)	*^1^H-NMR*	Propionate, acetate, succinate, putrescine, tyrosine Lactate, pyruvate, butyrate, 4-hydroxybenzoate, 4-pyridoxoate	[78]
*Medication- related osteonecrosis of the jaw* (17 v. 18)	*CE-MS*	Hypotaurine	[79]
*Parotid gland tumour* (36 v. 23)	*^1^H-NMR*	Alanine, threonine, leucine, serine, valine Formate, succinate	[80]
*Obesity* (22 v. 46)	*HPLC-MS/MS and GC-MS*	Creatinine, phosphate, cadaverine, dipeptides, putrescine	[81]
*Paediatric obesity* (18 v. 23)	*GC-MS*	Palmitic acid, myristic acid, urea, N-acetyl galactosamine	[82]
*External apical root resorption in orthodontic therapy* (8 v. 11)	*^1^H-NMR*	Butyrate, urea, glucose, formate, fumarate	[83]
